# Detection of metabolic change in glioblastoma cells after radiotherapy using hyperpolarized ^13^C‐MRI

**DOI:** 10.1002/nbm.4514

**Published:** 2021-05-03

**Authors:** Tatsuya Kawai, Jeffrey R. Brender, Jennifer A. Lee, Tamalee Kramp, Shun Kishimoto, Murali C. Krishna, Philip Tofilon, Kevin A. Camphausen

**Affiliations:** ^1^ Radiation Oncology Branch National Cancer Institute Bethesda Maryland USA; ^2^ Department of Radiology Nagoya City University Graduate School of Medical Sciences Nagoya Japan; ^3^ Radiation Biology Branch National Cancer Institute Bethesda Maryland USA

**Keywords:** ^13^C‐MRI, dynamic nuclear polarization, glioblastoma, glioma stem‐like cell, hyperpolarized MRI, in vivo glioblastoma, radiation therapy, tumor metabolism

## Abstract

Dynamic nuclear polarization (DNP) of ^13^C‐labeled substrates enables the use of magnetic resonance imaging (MRI) to monitor specific enzymatic reactions in tumors and offers an opportunity to investigate these differences. In this study, DNP‐MRI chemical shift imaging with hyperpolarized [1‐^13^C] pyruvate was conducted to evaluate the metabolic change in glycolytic profiles after radiation of two glioma stem‐like cell‐derived gliomas (GBMJ1 and NSC11) and an adherent human glioblastoma cell line (U251) in an orthotopic xenograft mouse model. The DNP‐MRI showed an increase in Lac/Pyr at 6 and 16 h after irradiation (18% ± 4% and 14% ± 3%, respectively; mean ± SEM) compared with unirradiated controls in GBMJ1 tumors, whereas no significant change was observed in U251 and NSC11 tumors. Metabolomic analysis likewise showed a significant increase in lactate in GBMJ1 tumors at 16 h. An immunoblot assay showed upregulation of lactate dehydrogenase‐A expression in GBMJ1 following radiation exposure, consistent with DNP‐MRI and metabolomic analysis. In conclusion, our preclinical study demonstrates that the DNP‐MRI technique has the potential to be a powerful diagnostic method with which to evaluate GBM tumor metabolism before and after radiation in the clinical setting.

## Graphical abstract

Orthotopic brain tumor mice were subjected to ^13^C‐MRI using hyperpolarized [1‐13C] pyruvate to investigate the effect of radiation exposure on glucose metabolism by measuring lactate‐to‐pyruvate ratio in the tumor. Tissue sampling was conducted independently to quantify the lactate concentration and LDHA expression by metabolomic and immunoblot analyses, respectively. These indicated an acceleration of lactate‐to‐pyruvate conversion in one of the stem‐like cell‐derived gliomas after an irradiation during a 24‐h period of time.

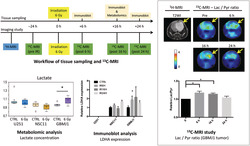

**Abbreviations used**
CSI: chemical shift imaging
DNP: dynamic nuclear polarization
GBM: glioblastoma multiforme
GSC: glioma stem‐like cell
HC: hierarchical cluster
Lac/Pyr ratio: lactate‐to‐pyruvate ratio
LDHA: lactate dehydrogenase A; MRI, magnetic resonance imaging
ROI: region of interest

## 1 INTRODUCTION

Glioblastoma, also known as glioblastoma multiforme (GBM), is the most common and deadly primary brain cancer, and is notoriously resistant to treatment due in part to extensive cellular and molecular heterogeneity, which leads to variable and short‐lived responses to most therapeutic agents.^1^ Currently, the standard of care for GBM consists of surgical resection, temozolomide and radiation therapy.^2^ However, despite this combination, the median survival for GBM remains approximately 15 months.^3^ Since the addition of temozolomide in 2005, no significant advancements have been made to the clinical treatment of GBM, thus, a greater understanding of the response of GBM to the current standard therapy is still needed.

It is well known that the cellular metabolism of cancer is abnormal, providing many potential novel targets for the treatment of GBM.^4^ The Warburg effect, in which there is a shift from oxidative phosphorylation to the lactate producing aerobic glycolysis, is the most significant and well‐studied phenomenon in tumor metabolism. This shift occurs even in the presence of oxygen and is believed to accommodate the increased demand for raw materials.^5^ The excessive lactate production of this shift has been correlated to increases in tumor migration, immune evasion and resistance to treatment including radiation therapy.^6^, ^7^, ^8^ With regard to such a metabolic shift, the contribution of glioma stem‐like cells (GSCs) to the resistance against treatments has recently gained attention because of their unique metabolic profiles.^9^, ^10^ Peng et al. suggested a relationship between the number of cells that presented a GSC marker and its aggressiveness.^11^ Indeed, increased glycolysis and lactate production has been observed in the clinic, spurring a larger effort into targeting GBM metabolism to improve therapeutic outcomes.^12^, ^13^ With this increased emphasis on tumor metabolism, methods to analyze the metabolic progression and response of tumors becomes paramount to the success of these metabolic therapies in the clinic.

Hyperpolarized magnetic resonance imaging (MRI) using dynamic nuclear polarization (DNP) is an emerging imaging technique that enables the observation of specific enzymatic reactions in tumors by detecting the enhanced signal of ^13^C‐labeled substrates that have been hyperpolarized under far‐beyond thermal equilibrium conditions.^14^ Hyperpolarized ^13^C‐MRI has the inherent ability of MRI to encode both spatial and spectral information that makes it possible to distinguish between the injected tracer and its downstream metabolic products. This enables metabolic pathways to be monitored over multiple enzyme‐regulated steps, potentially revealing sensitive and specific indicators of disease. Currently, the number of metabolic products, the expression levels of the correlated enzymes, and the enzymatic activities of orthotopic models of GBM following radiation, have not been previously shown. In the present research, a series of ^13^C‐MRI studies were conducted to evaluate the changes in metabolism of three different GBM tumors, including GSC‐derived tumors over a 24‐h period following a single dose of radiation. We show an elevation of the lactate‐to‐pyruvate ratio as well as a correlation between the amount of lactate and the expression of lactate dehydrogenase A (LDHA) in only one of the GSC‐derived tumors, where the other two tumors tested showed no changes. These results support the feasibility of ^13^C‐MRI using the DNP technique to fulfill the currently unmet need to noninvasively monitor case‐specific tumor metabolism and its response to therapies.

## 2 MATERIALS AND METHODS

### 2.1 Cell lines

The U251 human GBM cell line was obtained from the Division of Cancer Treatment and Diagnosis Tumor Repository (National Cancer Institute), grown in Dulbecco's Modified Eagle Medium (DMEM) supplemented with 10% fetal bovine serum (Invitrogen), then maintained in an atmosphere of 5% CO_2_/95% air at 37°C. Regarding the neurosphere‐forming GCSs: NSC11 was kindly provided by Dr Frederick Lang, MD Anderson Cancer Center, and GBMJ1 was generated at the Moffitt Cancer Center.^15^, ^16^ Neurospheres were maintained in a medium consisting of DMEM/F‐12 (Invitrogen, Carlsbad, CA, USA), B27 supplement (1x; Invitrogen), and human recombinant basic fibroblast growth factor and epidermal growth factor (50 ng/mL each; R&D Systems, Minneapolis, MN, USA). All cultures were maintained at 37°C in an atmosphere of 5% CO_2_/7% O_2_.^17^ NSC11 and GBMJ1 cells were cultured less than 2 months after resuscitation. Both U251 and GCSs tested negative for mycoplasma contamination by PCR analysis.

### 2.2 Generation of intracranial xenografts

Athymic nude mice (nu/nu, female; National Cancer Institute Animal Production, Frederick, MD, USA) aged 10–12 weeks were used. To implant tumor cells, the mice were anesthetized using isoflurane gas then placed in a small animal stereotactic apparatus (Stoelting, Wood Dale, IL, USA). To generate NSC11 and GBMJ1 tumors, neurospheres were dissociated into single‐cell suspensions; 1 x 10^5^ cells were then injected in a total volume of 2.5 μL at 1.0 mm anterior and 2.0 mm lateral to the bregma to a depth of 3.5 mm at a rate of approximately 1 μL/minute as previously described.^16^, ^18^ To generate a U251 tumor, 2.5 x 10^5^ cells were injected in a total volume of 2.5 μL. The mice were observed daily until the onset of neurologic symptoms (morbidity). All experiments were performed as approved by the Institutional Animal Care and Use Committee.

### 2.3 Coil design

MRI imaging was performed on a 3‐T MRS3000 magnet (MR Solutions, Guildford, UK) equipped with 280 mT/m gradients (GS‐150 gradient amplifier, Performance Controls Inc., Montgomeryville, PA, USA) and modified to have ^13^C capability. Scans were acquired using a dual‐tuned ^1^H/^13^C coil built in‐house consisting of a 35‐mm single‐loop linearly polarized surface coil on the outside for the proton channel and a 20‐mm transmit receive saddle coil in quadrature on the inside surrounding the head for the carbon channel.

### 2.4 ^1^H‐MRI imaging

At 1‐week intervals, the tumor was localized and the anatomy was defined by Fast Spin Echo T_2_‐weighted images (T2WI; TR/TE: 2500/68 ms; slice thickness: 1 mm; field of view: 28 x 28 mm; in‐plane resolution: 256 x 256, voxel size 0.11 × 0.11 × 1 mm^3^, echo train length of 8) using a 1‐T scanner (Bruker; Billerica, MA, USA). Mice that met the following criteria were chosen for further experimentation as described below: (1) the tumor was located within the brain parenchyma with a volume of 25–50 mm^3^ (U251 xenografts) or 30–75 mm^3^ (NSC11 and GBMJ1 xenografts); (2) no evidence of necrosis or hemorrhages were seen on T2WI. U251 tumors were evaluated at a smaller size to avoid the development of necrosis, which could interfere with the subsequent studies, including the ^13^C‐MRI study.

### 2.5 Irradiation

For in vivo studies, a single dose of 6‐Gy total‐brain irradiation was delivered under isoflurane anesthesia using another 320 kV X‐ray machine (Precision X‐Ray Inc.) with a set voltage and currency of 300 kV and 10 mA at a dose rate of 2.16 Gy/minute. Nonirradiated control mice were mock irradiated.

### 2.6 ^13^C hyperpolarized MRI experiments

**FIGURE 1:**
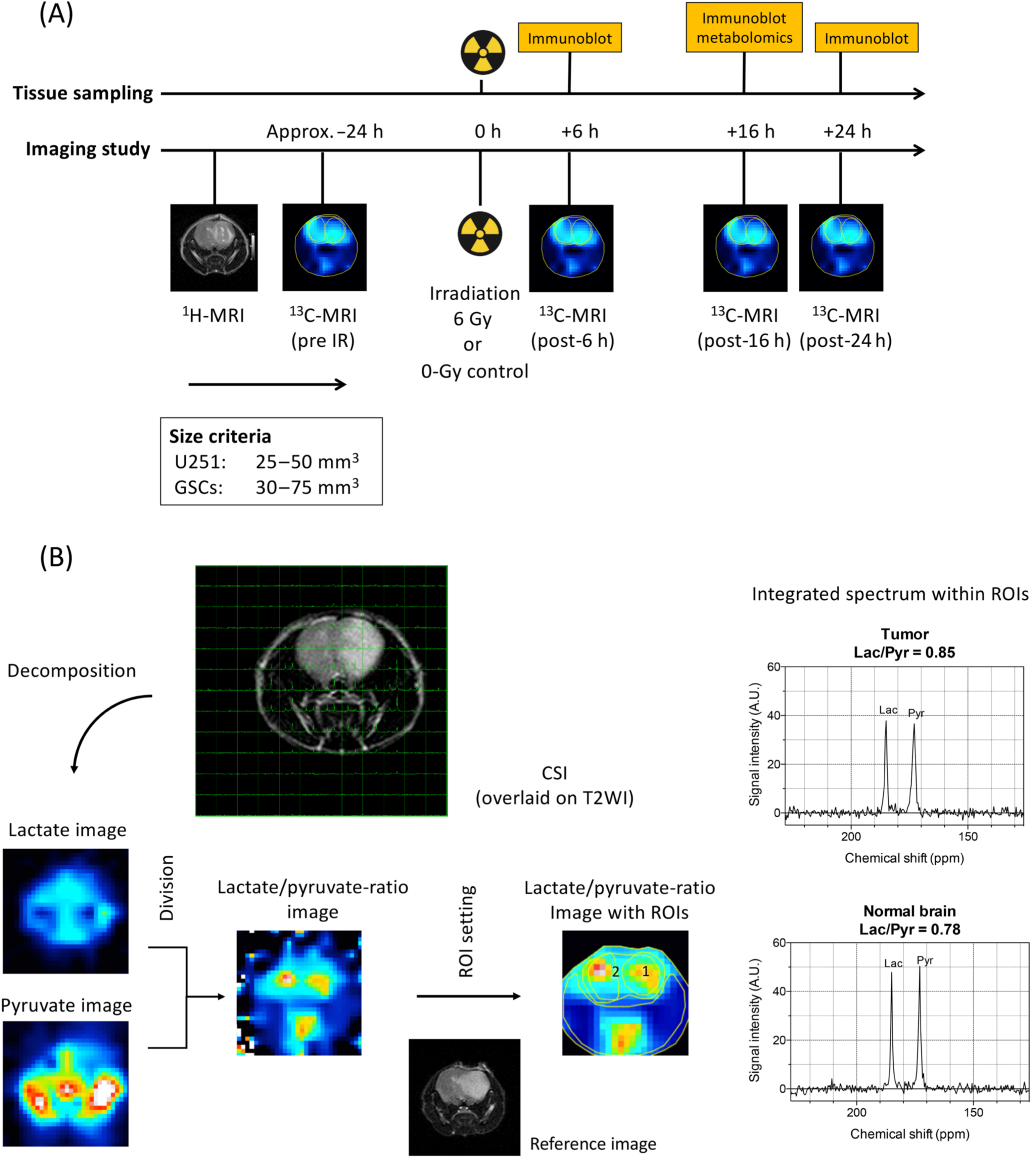
The workflow of in vivo ^13^C‐MRI scanning and tissue sampling. (A) Using a 1.0 T‐MRI, the size and imaging characteristics of orthotopic brain tumor were monitored every week after implantation. The ^13^C‐MRI study was initiated using 3 T‐MRI once the tumor meets the following criteria: tumor volume within 25–50 mm^3^ or 30–75 mm^3^ for U251 or GSCs (NSC11 and GBMJ1), respectively. For imaging study, the first ^13^C‐MRI was performed at 18–24 h before irradiation. After 6‐Gy irradiation or 0‐Gy sham irradiation was given, consecutive ^13^C‐MRI scans were performed at 6, 16 and 24 h. Any tumors with hemorrhage, necrosis or cystic lesion larger than 1 mm in diameter were excluded. Tumor volume was calculated by the following formula: length x width x height x 3.14 x 1/6. The tumor tissue was harvested at 6, 16 and 24 h after irradiation, or 16 h after irradiation for immunoblot analysis or metabolomics analysis, respectively. (B) After the acquisition of CSI (left‐upper panel), CSI data were decomposed to a series of 14 x 14‐pixel CSI data, followed by being converted to 42 x 42‐pixel images by bilinear spatial interpolation. The acquired single time point CSI data were processed by MATLAB software that reconstructed a set of 256 intensity images, each of them corresponding to each chemical shift ranging from 128 to 255 ppm. Among them, two sets of five consecutive images that included the lactate and pyruvate peak (183 and 171 ppm, respectively) were selected and fused by ImageJ software using the ‘z‐projection’ function to create the lactate and pyruvate images (lower left panels). A Lac/Pyr‐ratio image was then calculated from the lactate and pyruvate images. The ROIs were determined on the tumor and the contralateral normal brain tissue using a T2WI as a reference. The Lac/Pyr ratios at each ROI were calculated from the signal intensity plots on the corresponding region (right panel). CSI, chemical shift imaging; GSC, glioma stem‐like cell; Lac/Pyr‐ratio, lactate‐to‐pyruvate ratio; ROI, region of interest

Hyperpolarized ^13^C MRI studies were performed 18–24 h before and 6, 16 and 24 h after irradiation using a 3‐T scanner (MR Solutions) (Figure 1A). Five animals were imaged for each tumor type. For the localization of the tumor, T2WI was acquired before each hyperpolarized experiment; 30 μL of pyruvic acid containing 15 mM of OX063 (trityl radical compound) and 2.5 mM of the gadolinium chelate were polarized at 3.35 T and 1.4 K in a Hypersense DNP polarizer (Oxford Instruments, Abingdon, UK), according to the manufacturer's instructions. After 1–1.5 h, the hyperpolarized sample was rapidly dissolved and injected (approximately 1.15 mmol/kg) through the tail vein cannula as previously reported.^19^ ^13^C two‐dimensional spectroscopic images were acquired 30 s after the start of the pyruvate injection, with a 28 × 28 mm^2^ FOV in an 8‐mm thickness slab, a matrix size of 14 × 14, a spectral width of 3333 Hz and a repetition time of 75 ms, using a sinc excitation pulse with a flip angle of 10°. The total time required to acquire each image was 19.2 s. During acquisition, the body temperature was kept in the range of 36.0 to 36.9°C. Image processing was performed using MATLAB (MathWorks, Natick, MA, USA) and ImageJ, as described in Figure 1B. Because lactate‐to‐pyruvate ratio (Lac/Pyr ratio) represents the degree to which pyruvate is converted to lactate in a given time, the Lac/Pyr ratio map was created from a combination of chemical‐shift imaging (CSI) datasets corresponding to lactate and pyruvate signals (183 and 171 ppm, respectively). To calculate the Lac/Pyr ratio, regions of interest (ROIs) were determined around the tumor and contralateral normal brain, and a histogram of the chemical shift in each ROI was created and the areas under the curve at each peak, corresponding to lactate and pyruvate, were calculated.

### 2.7 Tumor tissue collection

For metabolomic and immunoblot analyses, tumor tissue was dissected from the mouse brain at specific time points after irradiation (16 h for metabolomic analysis; 6, 16 and 24 h for immunoblot analysis), as shown in Figure 1A. Tumor dissection was performed as previously described.^20^, ^21^ Briefly, the mouse was anesthetized with isoflurane, followed by an injection of analgesics (meloxicam). The vessel bed was infused by cardiac puncture using cold saline followed by 4% paraformaldehyde immediately after the rib cage opening to achieve a rapid and uniform fixation before changes in response to hypoxia began. The brain was excised and tumor tissue was carefully dissected from the brain using MRI findings as a reference, then snap‐frozen using liquid nitrogen and stored in a deep freezer at −80°C. The excision procedure took approximately 10 min from the skin excision to the snap‐freezing in the liquid nitrogen.

### 2.8 Metabolomic analysis

Metabolomic studies were conducted at Metabolon Inc. (Morrisville, NC, USA). A total of 36 samples were examined for global metabolic profiles in the three different GBM cell lines under control and irradiation‐treated conditions (N = 6 per tumor type, each treatment group). The metabolic profiling analysis combined three independent platforms to obtain broad coverage of the biochemical classes. This included ultrahigh performance liquid chromatography/tandem mass spectrometry (UHPLC/MS‐MS2) optimized for basic species, UHPLC/MS‐MS2 optimized for acidic species, and gas chromatography/mass spectrometry. Detailed descriptions of these platforms, including instrumentation configurations and conditions, data acquisition, and software approaches for data handling, were previously described in detail.^22^, ^23^

### 2.9 Immunoblot analysis

Frozen tumor tissue prepared as described in the previous section was homogenized (Biomasher II) in lysis buffer including protease/phosphatase inhibitors (Cell Signaling) and incubated for 30 min at 4°C; protein was subjected to immunoblot analyses as previously described.^24^ N = 4 for U251 (control, 6, 16 and 24 h), N = 5 for NSC11 and GBMJ1 (control, 6 and 24 h), and N = 4 for NSC11 and GBMJ1 (16 h). Anti‐LDHA and anti‐β‐tubulin were obtained from Cell Signaling. Donkey anti‐rabbit IRDye conjugated secondary antibody (LICOR Biosciences) was used for band detection using the Odyssey CLx Imaging System (LICOR Biosciences). For protein quantification, the intensity of each band was calculated using ImageJ.

### 2.10 Statistical analysis

For the ^13^C‐MRI study, we employed the relative Lac/Pyr ratio, which was calculated by dividing the Lac/Pyr ratio in the ROI at the tumor by the ratio at the in‐plane contralateral normal brain region, to semiquantify the pyruvate‐to‐lactate conversion. This method followed a previous report (Radoul et al.^25^) that was suitable for the single‐time point CSI, which showed that some degree of variability in the concentration of hyperpolarized ^13^C probe potentially affecting reproducibility was inevitable due to fluctuations in hyperpolarizing efficiency and injection rate at the time of MRS acquisition. In the metabolomic analysis, Welch's two‐sample t‐test was used following log transformation and imputation of missing values with the minimum observed value for each compound to identify biochemicals that differed significantly between experimental groups. Statistical analysis of log‐transformed data was conducted using “R” (http://cran.r-project.org/), which is a freely available, open‐source software package. Multiple comparisons were accounted for by estimating the false discovery rate using q values. A hierarchical cluster analysis and principal component analysis were carried out as well as a random forest analysis on untransformed data. For the immunoblot analysis, two‐way ANOVA (α = 0.05) was used along with Tukey's multiple comparison test to detect the difference in means of the quantified LDHA protein in the four different time points with statistical significance.

## 3 RESULTS

### 3.1 [1‐^13^C] pyruvate imaging

**FIGURE 2:**
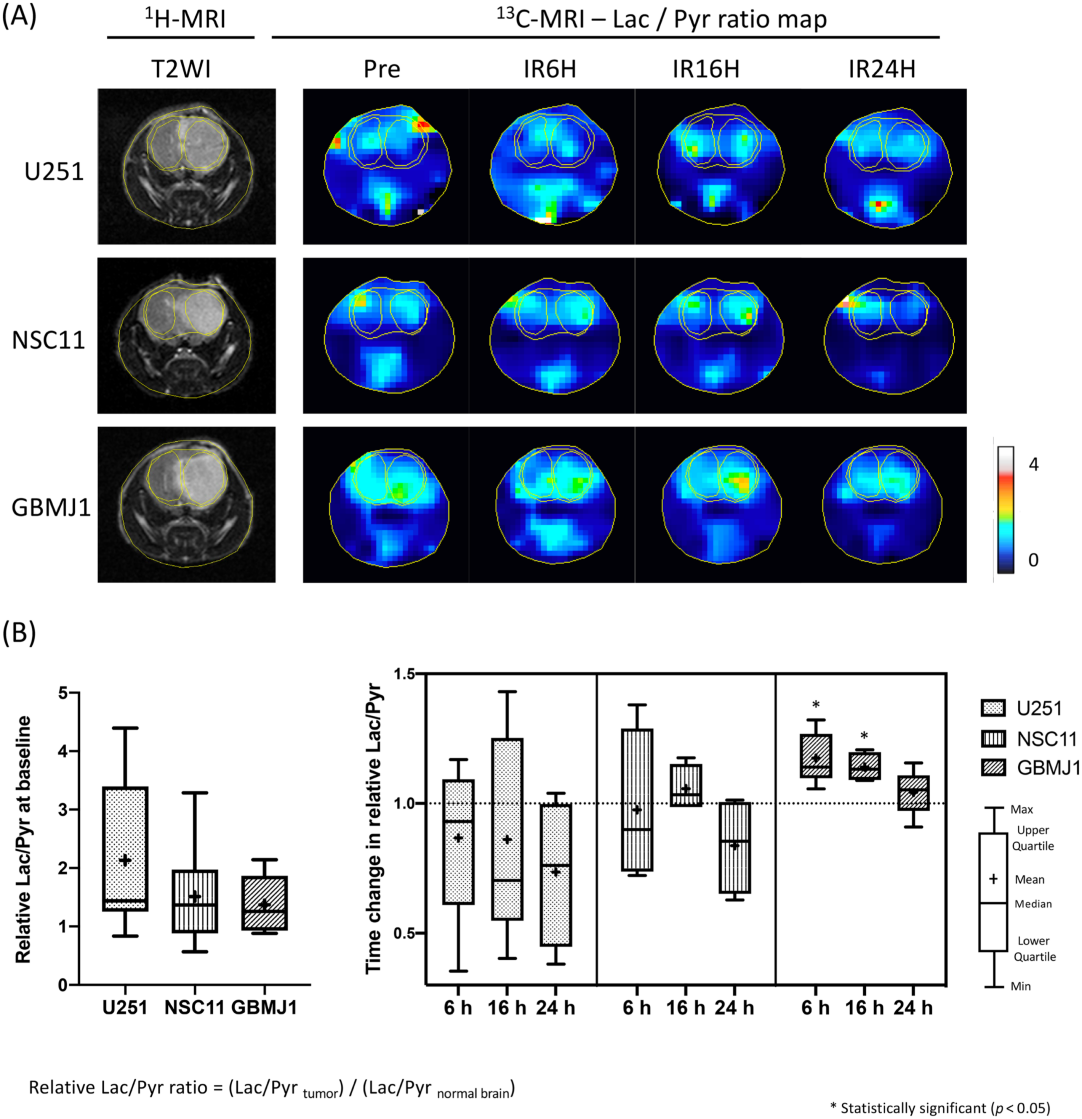
^13^C‐MRI displays the difference of the response in Lac/Pyr ratio to 6‐Gy irradiation among the three types of tumors. (A) A series of ^13^C‐MRI studies were performed 18–24 h before and 6, 16 and 24 h after irradiation (pre, 6 h, 16 h and 24 h, respectively) when the tumor grew to meet the criteria on T2WI, as described in the Materials and Methods section. ^13^C‐Lac/Pyr maps of the GBMJ1 tumor show increased Lac/Pyr ratio at 6 and 16 h after irradiation. (B) The Lac/Pyr ratio was separately calculated within each ROI at the tumor and the contralateral normal brain. The relative Lac/Pyr ratio at the tumor was calculated by dividing the Lac/Pyr ratio on the tumor by the one in the contralateral normal brain. Box‐and‐whisker plots of the relative Lac/Pyr ratios before irradiation (left panel) and those after irradiation standardized by those before irradiation (right panel) show a significant increase in the GBMJ1 tumor at 6 and 16 h (1.18 +/− 0.04 and 1.14 +/− 0.03, respectively; mean +/− SEM) (B). Each group contains five independent animals

To evaluate the differences in the metabolism of xenograft orthotopic glioma tumors, a series of ^13^C‐MRI studies was carried out 24 h before and 6, 16 and 24 h after irradiation (Figure 1A). Each mouse had a standard ^1^H anatomic MRI followed by [1‐^13^C] pyruvate imaging. The baseline Lac/Pyr ratios relative to the contralateral normal brain in preirradiated tumors were 2.13 ± 1.30, 1.51 ± 0.86 and 1.37 ± 0.46 (mean ± SD) for U251, NSC11 and GBMJ1, respectively (Figure 2B, left panel). As shown in Figure 2A, the Lac/Pyr ratio in the GBMJ1 tumor increased compared with the contralateral normal brain at 6 and 16 h after 6‐Gy irradiation (displayed in the yellow and red color wash) whereas no apparent change was seen within the U251 or NSC11 tumors. A standardized Lac/Pyr ratio was calculated by dividing the tumor Lac/Pyr ratio by the ratio in the contralateral normal brain. Cumulative plots of the standardized Lac/Pyr ratios after irradiation showed a significant increase within the GBMJ1 tumor at 6 and 16 h after irradiation (18% ± 4% and 14% ± 3%, respectively; mean ± SEM) (Figure 2B, right panel). These data suggest that pyruvate and lactate can be visualized in an orthotopic GBM tumor and that the metabolic response following irradiation is not uniform across tumors.

### 3.2 Metabolomic analysis

**FIGURE 3:**
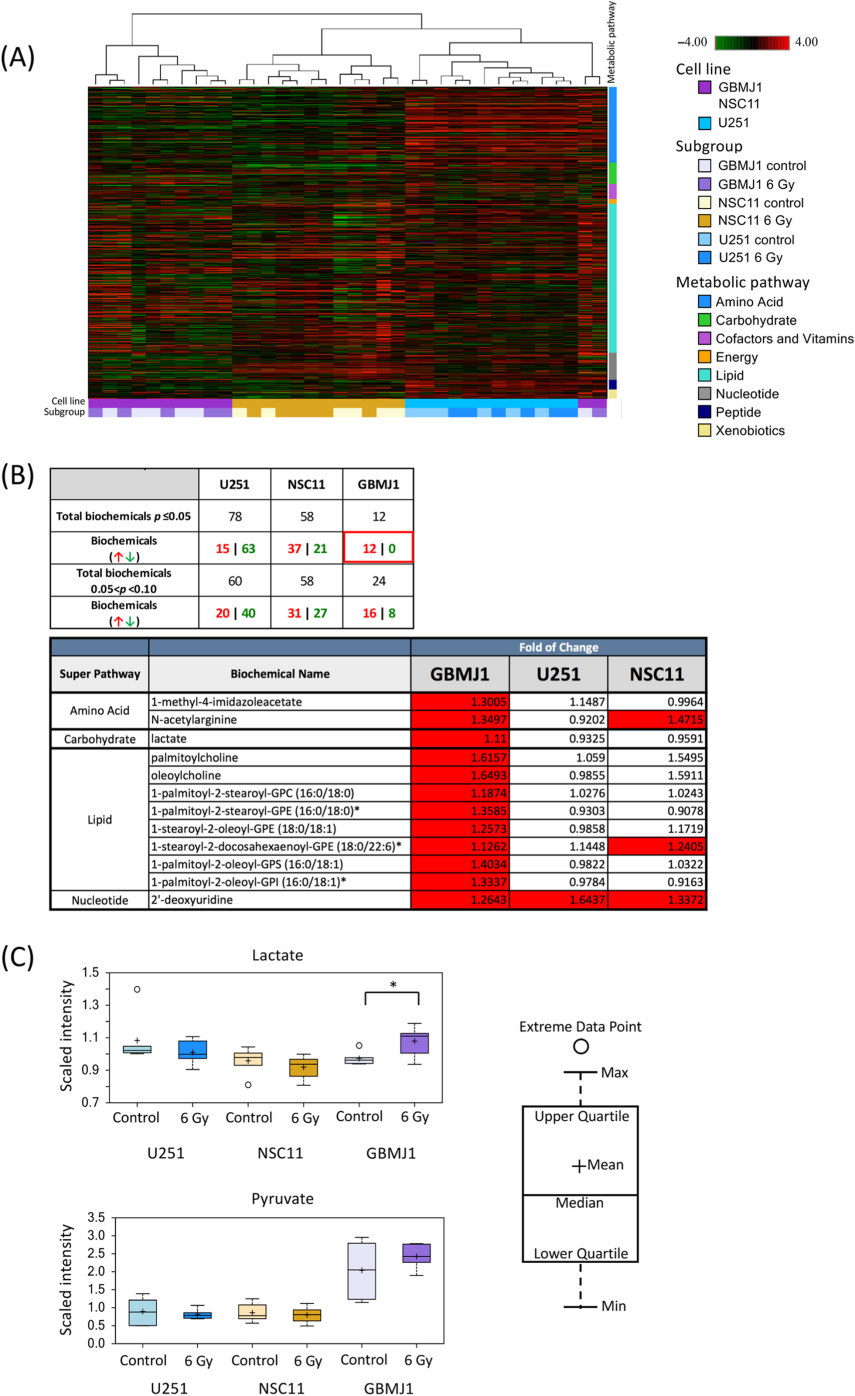
The metabolomic analysis shows an elevation of lactate in the GBMJ1 tumor. Metabolomic analysis was performed using the tumor tissue samples excised at 16 h after irradiation (N = 6 per each group). (A) The hierarchical cluster analysis shows significant separation among each type of the three tumors; however, radiation exposure does not result in significant separation from the control for each tumor. (B and C) Welch's two‐sample t‐test reveals 12 increased biochemical molecules that achieved statistical significance (*p* ≤ 0.05) in GBMJ tumors following irradiation. Among those molecules, a significant (1.11‐fold) increase in lactate in the GBMJ1 is found, whereas there is no significant change in the U251 and NSC11. While there is no significant difference in pyruvate between the control and irradiated groups in the GBMJ1, it is higher than the other tumors, both in the control and irradiated groups, respectively

To visualize the global metabolic profile of each tumor, samples were collected before and after irradiation, and total metabolite concentrations were determined by metabolomic analysis. The hierarchical cluster (HC) analysis showed a clean separation by cell line, whereas there was not a significant separation within tumors between the unirradiated and irradiated samples (Figure 3A, Figure S1A). Welch's two‐sample t‐test detected 12 molecules (Figure 3B, Figure S1B) that achieved statistical significance (*p* < 0.05) in the GBMJ1 tumors. Significant outliers for the U251 and NSC11 tumors are shown in Figure S1C. A significant increase (1.11‐fold) in lactate in GBMJ1 at 16 h after irradiation was measured, whereas no significant difference in lactate was observed in the U251 and NSC11 tumors (Figure 3C, upper panel). Although there was no significant difference in pyruvate between the control and irradiated groups in the GBMJ1, the overall amount of pyruvate was higher than the other tumors; the mean scaled intensities of pyruvate were 0.893, 0.857 and 2.038 for U251, NSC11 and GBMJ1, respectively, for the nonirradiated control tumors, and 0.814, 0.798 and 2.425, respectively, for the irradiated tumors (Figure 3C, lower panel). These data confirm that there is an increase in the lactate concentration in the GBMJ1 tumors that can be imaged using hyperpolarized ^13^C.

### 3.3 Immunoblot analysis

**FIGURE 4:**
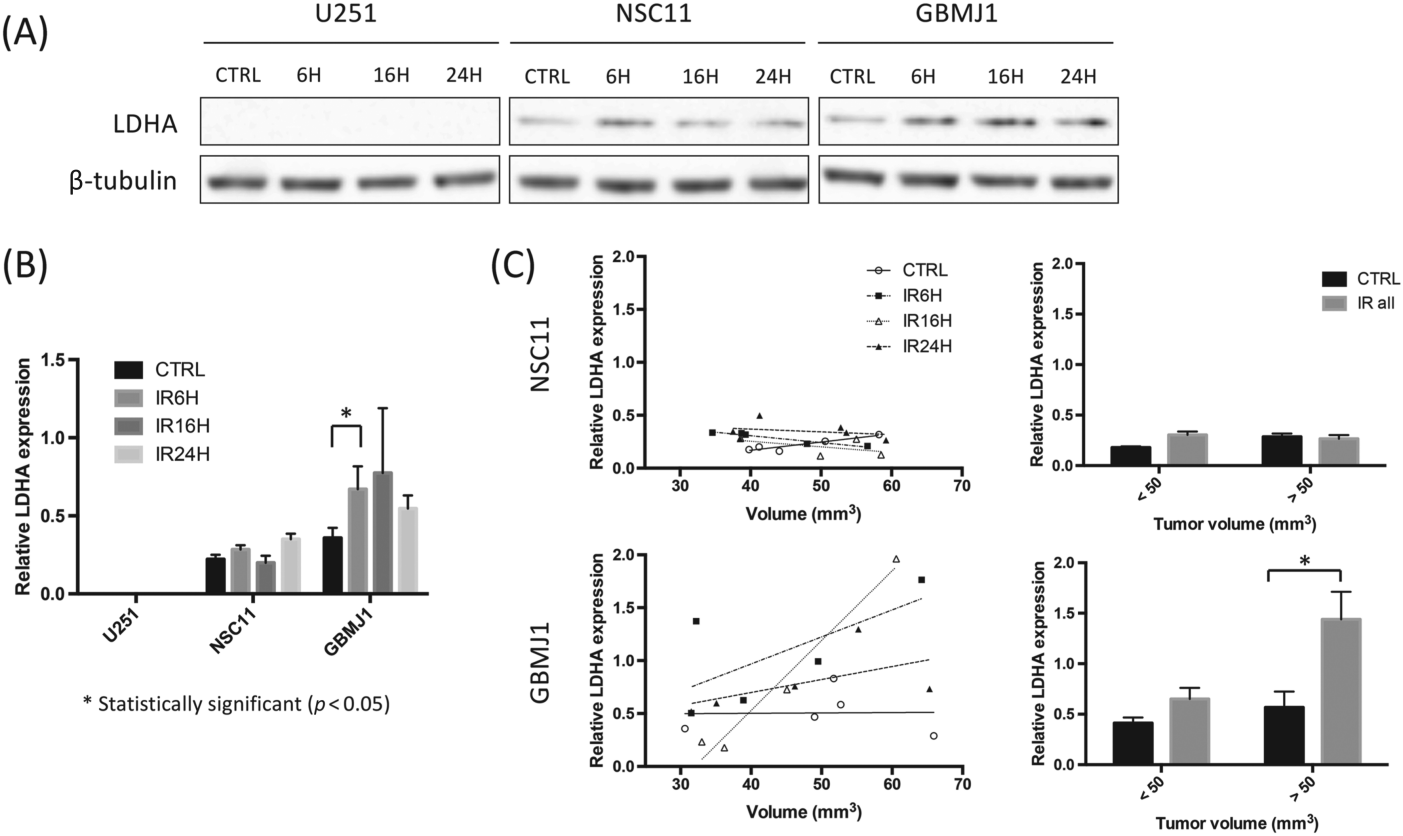
Immunoblot analysis shows the overexpression of LDHA in the GBMJ1 tumor in the orthotopic model treated with 6‐Gy irradiation. Immunoblot was performed with whole tumor homogenates of U251, NSC11 and GBMJ1 orthotopic tumors harvested at 6 and 24 h after 6‐Gy irradiation or 6 h after 0‐Gy sham irradiation (IR6H, IR24H and CTRL, respectively). The β‐tubulin protein was used as a loading control. Each treatment group contains four (U251), five (NSC11 and GBMJ1 for control, 6 and 24 h), or four (NSC11 and GBMJ1 for 16 h) independent tissue samples. (A and B) The basal protein expression level of LDHA is higher in GBMJ1 than in NSC11. The LDHA expression in the GBMJ1 tumor at 6 h after 6‐Gy irradiation is higher compared with the control group. LDHA protein is not detected in U251 tumors; bars, SE. * *p* < 0.05. (C) There is a positive correlation between the tumor volume and LDHA expression in the GBMJ1 after irradiation (lower panels), whereas no significant effect is observed in the NSC11 (upper panels)

To determine if there are changes in the primary metabolic enzyme LDHA that might correlate with the changes in lactate levels, immunoblot analysis using whole tumor homogenates of U251, NSC11 and GBMJ1 orthotopic tumors harvested at 0, 6, 16 and 24 h after 6‐Gy irradiation or at the same time period after 0‐Gy sham irradiation, were performed. As shown in Figure 4A,B, there was an increase in LDHA in the GBMJ1 tumors 6 h after irradiation. There was no LDHA detected in U251 tumors and minimal changes of LDHA levels in the NSC11 tumors after irradiation. When comparing the size of tumors to LDHA expression (Figure 4C), there was an increase in LDHA expression as the GBMJ1 tumors grew in size that was not seen in the NSC11 tumors. The tumor volumes for NSC11 and GBMJ1 were 46.8 ± 8.3 and 46.0 ± 12.0 cm^3^ (mean ± SD), respectively, and there were no significant differences among the treatment groups. These data further demonstrate that ^13^C in vivo imaging correlates to ex vivo metabolic profiling and may have a correlation to metabolic enzyme levels in our orthotopic GBMJ1 model.

## 4 DISCUSSION

With increasing interest in targeting tumor metabolism to circumvent treatment resistances associated with GBM, the identification of noninvasive methods to monitor the metabolic status of a tumor is of greater importance for clinical translation of these therapies. In this study, we investigated the use of MRI detection of hyperpolarized ^13^C‐labeled pyruvate to characterize the metabolic status of orthotopic GBM (U251) and GSC‐derived (NSC11 and GBMJ1) tumors before and after radiation therapy. We further validated our imaging results using protein and metabolomic analyses. Of the three tumor cell lines tested, we found that only GBMJ1 tumors had detectable changes in metabolism within a 24‐h period following a 6‐Gy dose of radiation. We observed an approximately 20% increase in the relative Lac/Pyr ratio of GBMJ1 tumors at 6 and 16 h postradiation followed by a recovery to its near preirradiation state at 24 h.

Metabolomic assays are a powerful technique to validate and build upon ^13^C‐MRI analyses. Our metabolomic evaluation of orthotopic tumors revealed that while baseline lactate levels among tumor types were similar, GBMJ1 tumors possessed a higher baseline level of pyruvate compared with U251 and NSC11 tumors. Although not explored in our study, this variation could be due to differences in the necrotic fraction, perfusion and composition of monocarboxylate transporters within each tumor type.^26^ With respect to treatment response, our metabolomic results mirrored that of our imaging analyses. While no changes in Lac/Pyr were detected for U251 and NSC11 tumors, GBMJ1 tumors showed a statistically significant increase in the Lac/Pyr ratio after irradiation. Because all tumors showed no significant changes in pyruvate levels following radiation, the increase in Lac/Pyr for GBMJ1 tumors could be attributed to the increase in lactate. Prior studies have shown that lactate production contributes to neurotoxicity and immune evasion allowing continued tumor growth and survival by lowering the pH in the tumor microenvironment.^27^, ^28^, ^29^, ^30^ As our studies focused on the immediate effects after radiation and were performed in immunocompromised animals, neither possibility could be evaluated, but present an interesting direction for future study.

While increases in LDHA following radiation have been observed in vitro, our work presents the first observation of upregulation of LDHA in response to radiation exposure in a GSC‐derived tumor using an orthotopic model.^31^ However, it remains unclear whether the difference in radiation response between our two GSCs could be explained by their inherent properties, such as MGMT (*O* ^6^‐methylguanine‐DNA methyltransferase) positivity. LDHA protein was not detected at any time in U251 tumors, consistent with its lowest LDHA expression among several glioma‐derived cell lines.^32^ One possibility is that LDHB, an isozyme of LDHA, takes the dominant role of pyruvate‐to‐lactate conversion in the U251 tumors instead of LDHA. There are many experimental factors that may have contributed to our novel findings. First, our study focused on the immediate effects of radiation on lactate and pyruvate levels, which were within 24 h after radiation. Previous studies have imaged the effects of radiation on metabolism in other tumor settings (SCCVII and HT‐29) or the effects of therapies such as temozolomide (TMZ) and everolimus on GBM metabolism, but evaluated time points at 24 h after therapy and beyond, showing decreases in Lac/Pyr ratios as a result of treatment or reduction in tumor burden.^19^, ^33^, ^34^ At 24 h we observed no changes in the Lac/Pyr ratio for all tumor types following 6‐Gy radiation, a finding similar to that of Day et al., who treated C6 bearing rats with a single dose of 15 Gy and showed a trend, but nonsignificant drop in Lac/Pyr at 24 h that became significant at 72‐h postradiation.^35^ This comparison highlights another important experimental consideration, which is the dose and frequency of the radiation used. A single dose of 6 Gy, like that used in our experiments, may not be large enough to impart a durable effect on tumor metabolism, particularly at the time points evaluated. Previous imaging studies demonstrating changes in tumor metabolism analyzed the effects of radiation doses of 10 Gy and above, again focusing on longer time points and fractionated treatments.^19^ The ability to noninvasively evaluate treatment response with hyperpolarized ^13^C‐MRI would be invaluable to treatment planning and fractionation of radiation.

Our study revealed a positive correlation between tumor volume and the degree to which 6‐Gy irradiation induced LDHA upregulation in the GBMJ1 tumor. Given that LDHA is one of the known transcripts regulated by HIF‐1, this mechanism might have contributed to our results as larger tumors tend to be more hypoxic and upregulate more HIF.^36^ With respect to targeting GBM metabolism to improve clinical outcomes, GBM cell lines have shown increased radiosensitivity when LDHA is or other glycolytic pathways are inhibited in vitro.^37^, ^38^, ^39^, ^40^ Given the lack of in vivo studies looking into the acute effects of therapies on tumor metabolism, our novel findings are motivation for further investigation into whether immediate changes in Lac/Pyr ratios and LDHA levels following radiation therapy could indicate a sensitivity or resistance to treatment.

Due to limits in spatial resolution, our ^13^C‐MRI study was limited to the evaluation of the pyruvate‐to‐lactate conversion in the tumor as a whole; however, investigating the distribution of Lac/Pyr ratio within the tumor, and its response to irradiation, including the contribution of innate immune cells, would be an interesting topic for future studies. Overall, our study demonstrated the utility of using hyperpolarized ^13^C‐MRI with the DNP technique to detect GBM tumor lactate levels following radiotherapy.

## AUTHOR DISCLOSURE INFORMATION

T. Kawai: None. J.R. Brender: None. J. Lee: None. T. Kramp: None. S. Kishimoto: None. M.C. Krishna: None. P. Tofilon: None. K.A. Camphausen: None.

## Supporting information


**Figure S1A.** Principle component analysis showed significant separation among each of the 3 tumor types, however, radiation‐treatment did not result in significant separation from the unirradiated groups.

**Figure S1B.** Welch's two‐sample t‐test detected 12 molecules that achieved statistical significance (p < 0.05) in the GBMJ1 tumors.

**Figure S1C.** Significant outliers for the U251 and NSC11 tumors.
