# Supplementary figures and images for: Detection of metabolic change in glioblastoma cells after radiotherapy using hyperpolarized 13C‐MRI
Source: NMR Biomed. 2021 May 3;34(7):e4514. doi: 10.1002/nbm.4514 (PMC8243917; doi:10.1002/nbm.4514)

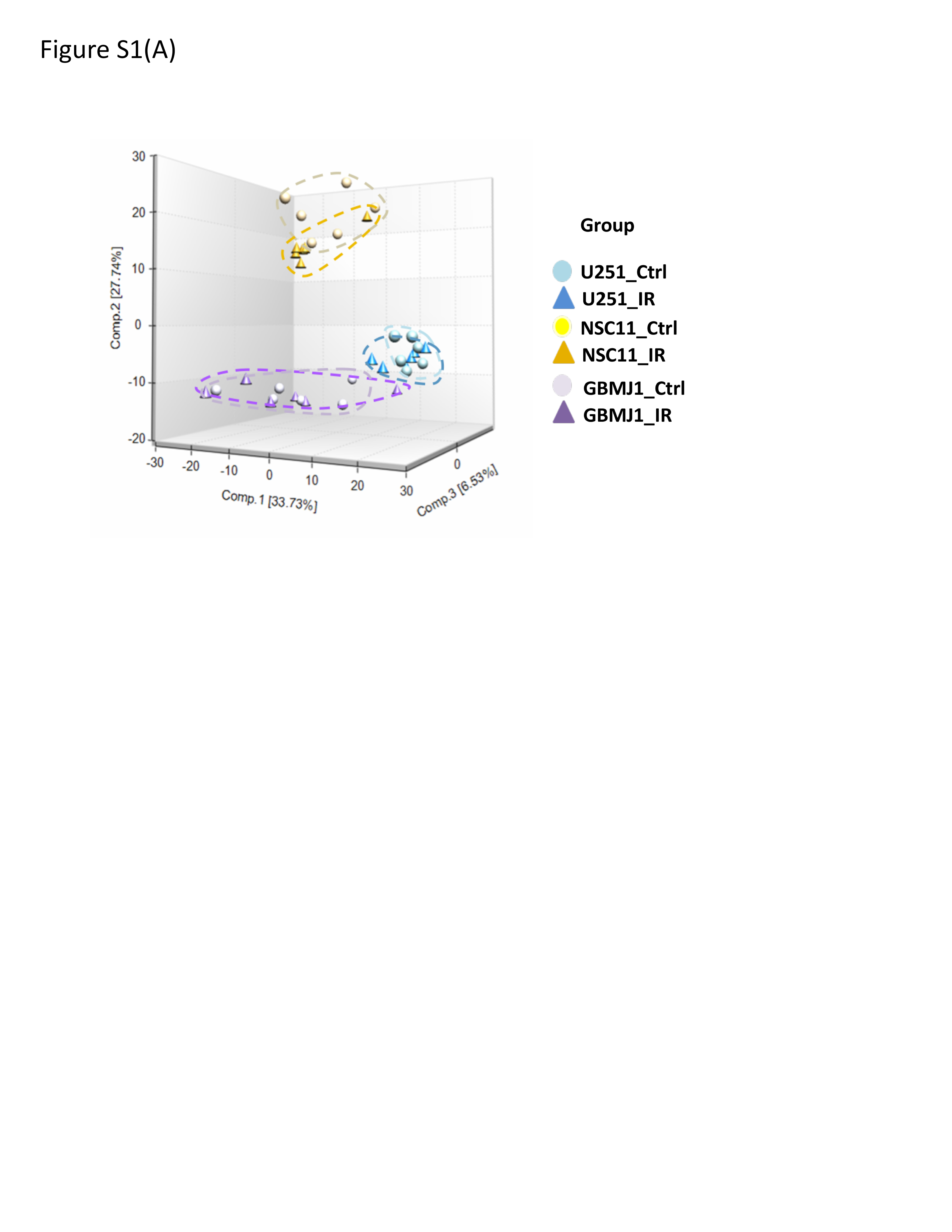

Supplement: Supplementary file 1 — Figure S1A. Principle component analysis showed significant separation among each of the 3 tumor types, however, radiation‐treatment did not result in significant separation from the unirradiated groups. [file NBM-34-e4514-s001.tif]

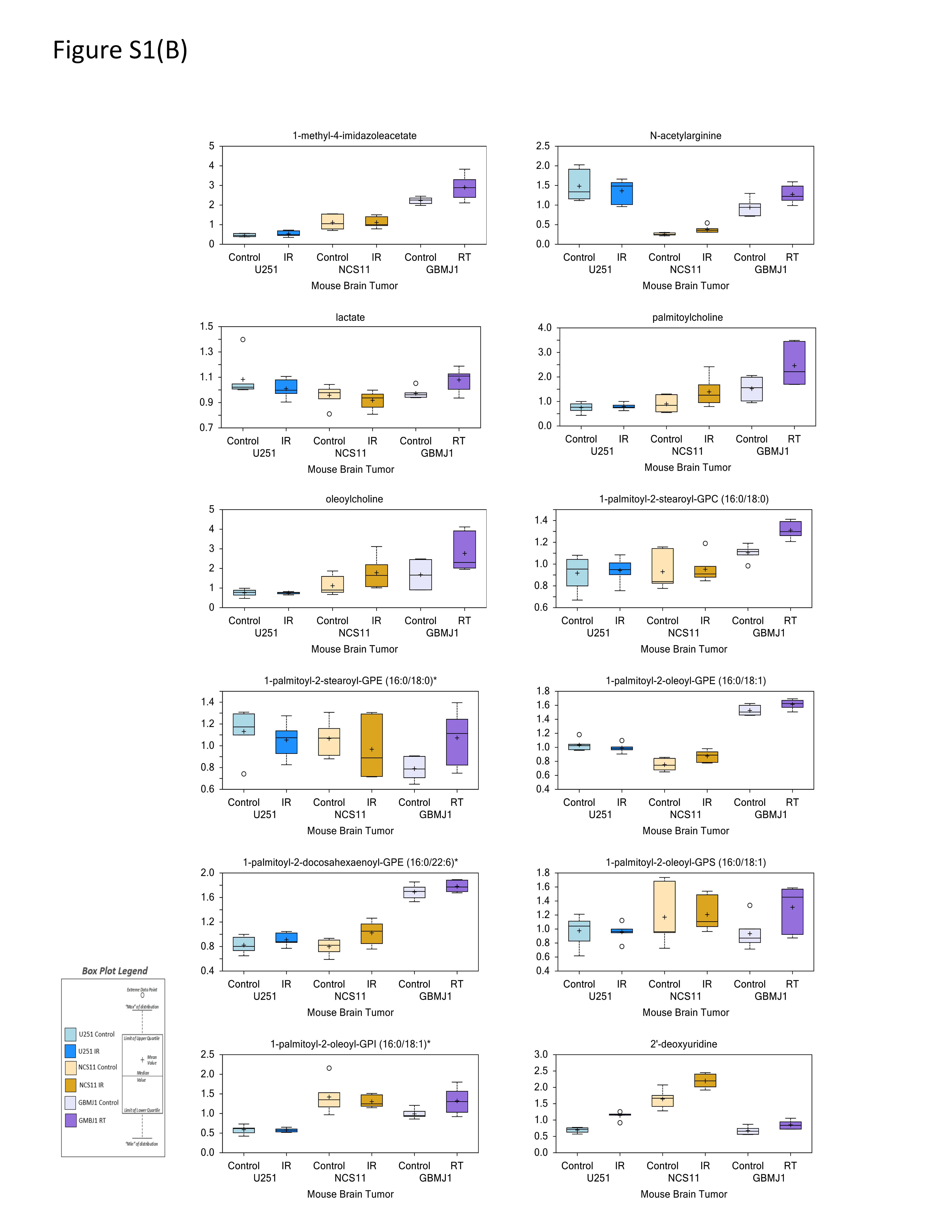

Supplement: Supplementary file 2 — Figure S1B. Welch's two‐sample t‐test detected 12 molecules that achieved statistical significance (p < 0.05) in the GBMJ1 tumors. [file NBM-34-e4514-s002.tif]

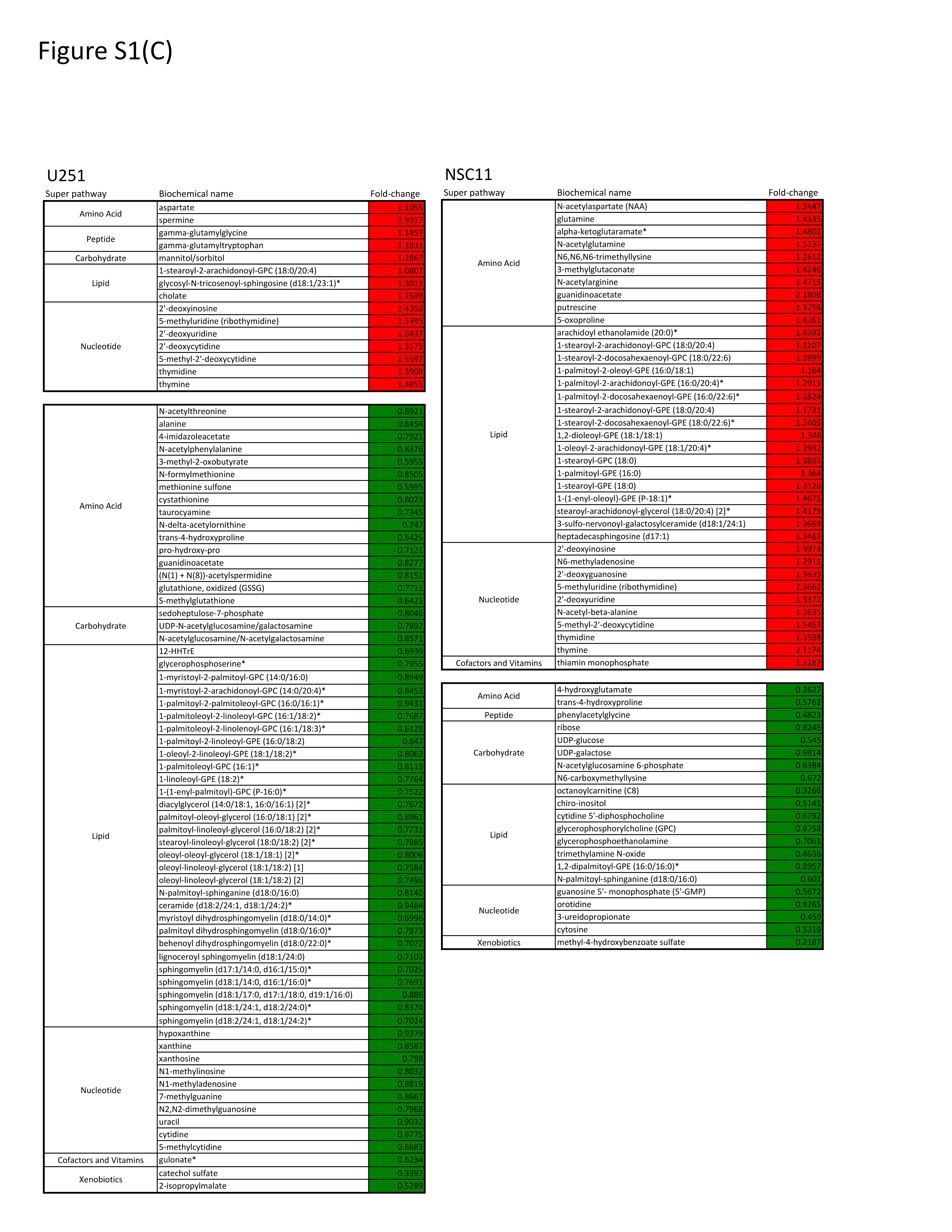

Supplement: Supplementary file 3 — Figure S1C. Significant outliers for the U251 and NSC11 tumors. [file NBM-34-e4514-s003.tif]
